# Hippocampal glucose uptake as a surrogate of metabolic change of microglia in Alzheimer’s disease

**DOI:** 10.1186/s12974-021-02244-6

**Published:** 2021-08-31

**Authors:** Hongyoon Choi, Yoori Choi, Eun Ji Lee, Hyun Kim, Youngsun Lee, Seokjun Kwon, Do Won Hwang, Dong Soo Lee, for the Alzheimer’s Disease Neuroimaging Initiative

**Affiliations:** 1 Department of Nuclear Medicine, Seoul National University Hospital, 101 Daehak-ro, Jongno-Gu, Seoul 03080 Seoul, Republic of Korea; 2 Department of Nuclear Medicine, Seoul National University College of Medicine, 101 Daehak-ro, Seoul, Jongo-Gu 03080 Republic of Korea; 3 Department of Molecular Medicine and Biopharmaceutical Sciences, Graduate School of Convergence Science and Technology, Seoul National University, 101 Daehak-ro, Jongno-Gu, Seoul 03080 Seoul, Republic of Korea

**Keywords:** Alzheimer’s disease, Microglia, Positron emission tomography, Hippocampus, Single-cell RNA-sequencing

## Abstract

**Abstract:**

**Background:**

Dynamically altered microglia play an important role in the progression of Alzheimer’s disease (AD). Here, we found a close association of the metabolic reconfiguration of microglia with increased hippocampal glucose uptake on [^18^F]fluorodeoxyglucose (FDG) PET.

**Methods:**

We used an AD animal model, 5xFAD, to analyze hippocampal glucose metabolism using both animal FDG PET and ex vivo FDG uptake test. Cells of the hippocampus were isolated to perform single-cell RNA-sequencing (scRNA-seq). The molecular features of cells associated with glucose metabolism were analyzed at a single-cell level. In order to apply our findings to human brain imaging study, brain FDG PET data obtained from the Alzheimer’s Disease Neuroimaging Initiative were analyzed. FDG uptake in the hippocampus was compared according to the diagnosis, AD, mild cognitive impairment, and controls. The correlation analysis between hippocampal FDG uptake and soluble TREM2 in cerebrospinal fluid was performed.

**Results:**

In the animal study, 8- and 12-month-old 5xFAD mice showed higher FDG uptake in the hippocampus than wild-type mice. Cellular FDG uptake tests showed that FDG activity in hippocampal microglia was increased in the AD model, while FDG activity in non-microglial cells of the hippocampus was not different between the AD model and wild-type. scRNA-seq data showed that changes in glucose metabolism signatures including glucose transporters, glycolysis and oxidative phosphorylation, mainly occurred in microglia. A subset of microglia with higher glucose transporters with defective glycolysis and oxidative phosphorylation was increased according to disease progression. In the human imaging study, we found a positive association between soluble TREM2 and hippocampal FDG uptake. FDG uptake in the hippocampus at the baseline scan predicted mild cognitive impairment conversion to AD.

**Conclusions:**

We identified the reconfiguration of microglial glucose metabolism in the hippocampus of AD, which could be evaluated by FDG PET as a feasible surrogate imaging biomarker for microglia-mediated inflammation.

**Supplementary Information:**

The online version contains supplementary material available at 10.1186/s12974-021-02244-6.

## Background

Alzheimer’s disease (AD) is the most common neurodegenerative disorder. AD is characterized by neuronal loss causing cognitive decline and the presence of amyloid beta deposits and neurofibrillary tangles in the brain [1, 2]. These pathological features serve as biomarkers to diagnose AD and are the basis for the ATN framework, a biomarker-based research (A, amyloid; T, phosphorylated tau; N, neurodegeneration) [3]. Although the stage of the AD continuum is diagnosed through ATN biomarker measurements, other biomarkers are needed to know prognosis and discover treatment targets of AD. Recently, neuroinflammation is not only a major factor with amyloid and tau in the pathology of AD, but also plays an important role in disease progression [4]. In particular, microglia play a key role in neuroinflammation associated with amyloid and tau accumulation, and synaptic dysfunction [5].

These pathologic characteristics are closely linked to the immune reaction, which is a complicated process in which microglia play a crucial role [4]. As microglia-mediated immune reactions are a dynamic process caused by various subtypes of microglia in the brain, recent studies have focused on the evaluation of the diversity of microglia potentially associated with the progression of AD pathophysiology [6, 7]. In general, reactive microglia play a role in the protection from CNS insults such as Aβ. Reactive microglia increase Aβ endocytosis and phagocytosis in AD, but these functions are degenerated due to chronic inflammatory conditions in AD [8]. A persistent inflammation induces changes of their phenotype that exhibits proinflammatory cytokines and nitric oxide release, increased proliferative responses, and decreased neurotrophic factors [9]. During this dynamic microglia-mediated inflammation, disease-associated microglia (DAM) have been defined as a subset of microglia that acquire a unique functional signature to attenuate the progression of neuronal loss in neurodegenerative disorders mediated by Triggering receptor expressed on myeloid cells-2 (TREM2) [5, 10]. TREM2 is a key surface receptor for the microglial response to neurodegeneration-related proteins, modulates phagocytosis, lipid metabolism, and metabolic shift to promote cell survival and restrict inflammation [11]. As a biomarker, the soluble form of TREM2 (sTREM2) measured in cerebrospinal fluid (CSF) recently receives attention in AD and other neurodegeneration disorders [12].

One of the key changes in microglia-mediated inflammation is metabolism, as immune cells depend on various metabolic pathways according to energy demands. The reactivity of the innate immune system is associated with a metabolic shift from oxidative phosphorylation (OXPHOS) to glycolysis, which is a rapid process of energy production. These active metabolic processes can be suppressed by chronic inflammatory processes due to broad defects in energy metabolism that underlie immunoparalysis [13]. This immunoparalysis induces phagocytic dysfunction of microglia in AD [14, 15].

Because of the important role of microglia in AD, a search for a biomarker for the functional evaluation of microglia has been attempted. In particular, molecular imaging methods based on positron emission tomography (PET) have been developed for targeting microglia. For example, [^11^C]PK11195 (1-[2-chlorophenyl]-N-methyl-N-[1-methyl-propyl]-3-isoquinolinecarboxamide) binds to translocator protein-18 kDa (TSPO), a marker of reactive microglia, has been used to evaluate microglial density in the brain [16, 17]. Although several modified radiotracers targeting TSPO have been recently studied, one of the limitations of TSPO PET includes the difficulty in reflecting recently emerging microglial reprogramming of dynamic changes according to neurodegeneration. Meanwhile, fluorodeoxyglucose (FDG), the most commonly used radiotracer for PET imaging, has been used to evaluate metabolic patterns of the brain in AD [18, 19]. As FDG PET represents regional glucose metabolism which is a universal feature of all cells, it is intrinsically difficult to differentiate the origin of FDG uptake and whether hypermetabolism on PET results from neurons or glia [20, 21]. For human AD studies, decreased glucose metabolism is mainly identified in some specific brain regions including the posterior cingulate, superior parietal and lateral temporal cortices [18, 19]. Although the results are controversial, a few studies have shown increased glucose metabolism in the hippocampus of AD [22–25]. However, the reason for regional differences in neuronal loss and metabolic change is still unknown, where neuronal loss is typically found in the hippocampus, and hypometabolism is found in the neocortex [26].

Here, we evaluated whether regional FDG uptake measured on PET could be used as a non-invasive tool for evaluating the status of neuroinflammation. We found that hippocampal glucose metabolism in AD is closely related to disease-associated metabolic reconfiguration of microglia and that hippocampal glucose metabolism using PET in the 5xFAD AD mouse model was correlated with microglial metabolism. Single-cell level analyses were used to support the characteristic metabolic changes in microglia of the hippocampus in AD. Furthermore, the human AD database of PET imaging and sTREM2 were analyzed to support our findings.

## Methods

### Animals and study designs

Male transgenic hemizygous 5xFAD mice (B6SJL-Tg (APPSwFlLon, PSEN1*M146L* L286V) 6799 Vas/Mmjax) were obtained from Jackson Laboratory (Bar Harbor, ME, USA). In order to obtain stable results, male mice were selected and tested because there are gender differences in the pathology of AD mouse models. Female wild-type (WT) mice were obtained from crossbreeding a C57BL/6 female x an SJL male. These mice were bred, and male transgenic hemizygous and male wild-type mice were used. They were kept under standard laboratory conditions (22–24 °C, 12-h light and dark cycle) with free access to water and standard feed. For FDG PET studies, a total of 19 5xFAD mice were used for FDG PET scans. FDG PET images were also acquired for age-matched WT mice (*n* = 21). PET scans were performed for 4, 8, and 12 months with ± 0.25 months.

For the ex vivo study of FDG uptake in microglia, nine 5xFAD mice and nine WT mice were sacrificed after FDG injection. The group of mice was divided according to age; 3.5, 7, and 13 months old. Single-cell RNA-sequencing (scRNA-seq) studies were performed on the brains of four mice (three 5xFAD and one WT mouse). Two-, 6-, and 9-month-old 5xFAD mice and a 3-month-old WT mouse were used to obtain hippocampal tissue. The animal study was approved by the Institutional Animal Care and Use Committee at Seoul National University (SNU-181018-6).

### Animal PET imaging

PET scans were performed on a dedicated small animal PET/CT scanner (eXplore VISTA, GE Healthcare, WI). All animals were fasted for at least 8 h before the start of the study. Animals were anesthetized with 2% isoflurane at 1 L/min oxygen flow for 5–10 min. FDG (10.5–11.7 MBq) was injected by an intravenous bolus injection. Static emission scans at 45 min after the injection were acquired. During the FDG uptake period, animals were awake and then anesthetized 10 min before PET/CT scans to reduce anesthetic effects on microglia [27]. Emission scans were acquired for 20 min. The energy window for the scan was 400–700 keV and images were reconstructed by a three-dimensional ordered-subsets expectation maximum (OSEM) algorithm with attenuation, random and scatter correction. The final voxel size was 0.3875 × 0.3875 × 0.775 mm.

### Animal PET analysis

Brain PET scans were manually examined for the quality control. All PET images were spatially normalized to the mouse brain template [28]. The spatial normalization was performed by Statistical Parametric Mapping (SPM8, University College of London, London, UK). To obtain hippocampal FDG uptake, we manually drew the right and left hippocampus on the brain template. The voxel counts were normalized to the global brain uptake in each PET image using a brain mask. Hippocampal FDG uptake was defined by using the relative value to global brain uptake.

### Cell FDG uptake study

After fasting for at least 8 h, 20.3–27.4 MBq FDG was injected into 5xFAD and age-matched controls intravenously. The hippocampus was isolated from the brain. Liver tissue was cut out and weighed at 40 minutes after injection. FDG uptake by the liver and microglia from the hippocampus was measured with a gamma counter (Packard Cobra II, GMI, NM, USA). Hippocampal FDG uptake values of microglia were estimated by the normalized value of counts per minute (cpm). FDG uptake values in the liver were calculated to cpm/mg. The FDG uptake values in microglia were divided by FDG uptake of the liver for each mouse. The FDG uptake values per cell were calculated using the total cell number.

### Cell sorting

The microglia were isolated according to the manufacturer’s instructions for magnetic-activated cell sorting (MACS) cell separation with mouse CD11b+ MicroBeads (Miltenyi Biotec, Bergisch Gladbach, Germany). The astrocytes were isolated according to the manufacturer’s instructions for MACS cell separation with mouse ACSA-2 (astrocyte cell surface antigen-2) MicroBeads (Miltenyi Biotec, Bergisch Gladbach, Germany). The neurons were isolated according to the manufacturer’s instructions for MACS cell separation with mouse neuron isolation kit (Miltenyi Biotec, Bergisch Gladbach, Germany).

### RT-PCR and quantitative PCR

RNA was isolated from sorted brain cells with TRIzol reagent (ambion) according to the manufacturer’s instructions. Reverse transcription of RNA was performed using a thermal cycler (Bio-Rad, T100). cDNA samples were amplified with primers. Then agarose gel electrophoresis was performed to analyze PCR products. For quantitative PCR, cDNA samples were mixed with SYBR green master mix (Takara) and loaded on an Applied Biosystems 7500. The mRNA levels of target gene, Trem2, were normalized to mRNA levels of each beta-actin. Primer information: Hexb: forward 5′GCTGTTGGTGAGAGACTCTGGA3′, reverse 5′GAGGTTGTGCAGCTATTCCACG3′; Snap25: forward 5′TTGGCTGAAACTATGTGAAATGGA3′, 5′ATGGTGATTAACAAGAGCCAGACG3′; Slc1a3: forward 5′TGTGCTTGTTTATGTCCCTACC3′, reverse 5′TCTCCTGCTGTGTTTTCTTCC3′; Trem2: forward 5′GGAACCGTCACCATCACTCT3′, reverse 5′ATGCTGGCTGCAAGAAACTT3′; Tyrobp: forward 5′GATTGCCCTGGCTGTGTACT3′, reverse 5′CTGGTCTCTGACCCTGAAGC3′; beta-actin: forward 5′AAGACCTCTATGCCAACACAGT3′, reverse 5′GCTCAGTAACAGTCCGCCTA3′

### Single cell dissociation from the hippocampus

For RNA sequencing, the Papain Dissociation System (Worthington, NJ, USA) was used to separate the hippocampus into single cells according to the manufacturer’s instructions. Dead Cell Removal Kit (Miltenyi Biotec, Bergisch Gladbach, Germany) was performed before loading to 10× to remove the dead cells.

For microglial dissociation, hippocampus tissue was incubated in 1 mg/mL papain, 5% D-(+)-trehalose dehydrate, 0.5 mM DL-2-amino-5-phosphonopentanoic acid, 100 units of DNase based on hibernate A without calcium and magnesium buffer in a 37 °C shaking incubator for 1 h. The cells were washed 3 times and then agitated using a pipette tip and passed through a 70-μm filter mesh. Discontinuous density gradient buffer (0.6 mg/mL ovomucoid inhibitor-albumin, 5% D-(+)-trehalose dehydrate, 0.8 mM DL-2-amino-5-phosphonopentanoic acid, 100 units of DNase based on hibernate A without calcium and magnesium buffer) was prepared in a tube. The cell layer suspension was carefully maintained on the gradient buffer, and then centrifuged at 900 rpm for 6 min and the dissociated cell pellet was resuspended.

### Single cell RNA-sequencing

The purified cells obtained from the hippocampus were sequenced using the Chromium single cell gene expression platform (10× Genomics). Approximately 1000–1500 cells from each dissected brain were directly loaded into each sample well following the manufacturer’s instructions. All samples were combined into droplets with barcoded beads. Manufacturer specifications were followed for generation of the barcoded libraries and then the samples were sequenced to an average depth of 40,000–60,000 reads on an Illumina HiSeq 2500 sequencer.

### Analysis of single cell RNA-sequencing

scRNA-seq samples were pseudoaligned to the ENSEMBL GRCm38 Mus musculus transcriptome using Kallisto, version 0.45.0 with the default option [29]. The reads were aligned to the index using Kallisto-bus [30]. Exonic counts for each barcode were estimated after the corrected BUS file with ‘bustools correct’ and ‘bustools sort’ using specifying “10× v2” chemistry as an option. After the estimation of gene counts using pseudoalignment, data were loaded using Seurat (version 3.0.0) [31]. All data were merged using canonical correlation analysis (CCA) as a part of the Seurat package. The cells were filtered for further analysis based on the following parameters: expression of at least 200 genes and at most 4000 genes to exclude cell duplets, and less than 10% of transcripts of mitochondrial genes. The transcript counts were log transformed with multiplication of scaling factor 10,000. Variable features (nfeatures = 2000) were identified based on a variance stabilizing transformation. Next, principal component analysis (PCA) was run on variable genes, and the first ten PCs were selected for clustering analyses. Cells were clustered using the FindClusters function in Seurat with default settings, resolution = 0.2. The marker genes of each cluster were identified by the FindAllMarkers function in Seurat. To visualize the transcript data, t-distributed stochastic neighborhood embedding (t-SNE) was used.

A subset of microglia was selected by specific clusters of microglia after the clustering of all cells. The subclusters of microglia were determined by another clustering process of the microglial subset. The highly variable genes (*n* = 2000) were reselected for the microglial subsets and then PCA was performed. The clusters of microglia were identified using the FindClusters function with resolution = 0.1.

### Estimation of glucose metabolism features of cells

The glucose metabolism features of cells were estimated by gene sets defined by Kyoto Encyclopedia of Genes and Genomes (KEGG) pathways. The specific metabolic pathways of mouse related to glucose metabolism were downloaded by GSKB for mouse [32]: ‘Glycolysis and Gluconeogenesis’ and ‘Oxidative phosphorylation’. A gene set of glucose transporters was selected (from Glut1 to Glut12) for use as another gene set related to glucose uptake. The cellular scores of the gene sets were estimated by the *AddModuleScore* function in Seurat.

### Human FDG PET and CSF soluble TREM2 data

In this study, human FDG PET data and CSF sTREM2 data were collected from the Alzheimer Disease Neuroimaging Initiative (ADNI) (http://adni.loni.usc.edu) database. To perform correlation analysis between FDG PET and CSF sTREM2, 98 patients: AD 27, normal controls (CN) 23, and mild cognitive impairment (MCI) 47, who underwent FDG PET, CSF sTREM2, and MRI were selected. The CSF sTREM2 was downloaded from the ADNI database (http://adni.loni.usc.edu). Among 47 MCI patients, 37 patients underwent more than 2 years of follow-up examinations. MCI patients were divided into two groups, MCI converters (MCI-C) and MCI non-converters (MCI-NC) according to follow-up diagnosis. Nineteen patients progressed to AD before 2 years, and they were defined as MCI-C. Eighteen patients did not progress to AD within 2 years and were defined as MCI-NC.

The ADNI (http://www.loni.usc.edu/) is a multicenter longitudinal study launched in 2003 as part of a $60 million, 5-year public-private partnership led by Principal Investigator Michael W Weiner. The purpose of the ADNI has been to develop and validate biomarkers for predicting the progression of MCI and/or early AD using serial MRI, PET, other biological markers, and clinical and neuropsychological data. Many coinvestigators from a broad range of academic institutions and private corporations work to find participants, and they have recruited participants from over 50 sites across the US and Canada. The institutional review boards of all participating institutions approved imaging and laboratory studies, and all participants signed a written informed consent form. For up-to-date information, see http://www.adni-info.org.

### Hippocampal FDG uptake evaluation for human PET

We evaluated FDG uptake of the hippocampus using partial volume correction. Input PET images were standardized to have the same voxel size (1.5 × 1.5 × 1.5 mm). A partial volume correction process was performed using a PVElab software package [33]. PET images were coregistered to T1-weighted MR images. Segmentation of MR data based on MNI atlas labeling was performed and voxelwise partial volume correction was performed using a method that combines the approach of modified Müller-Gärtner proposed by Rousset et al. [34]. Count normalization was performed based on global intensity of individual.

### Statistical analysis

The comparison of hippocampal FDG uptake between two groups, 5xFAD and WT mice, was performed using Mann-Whitney tests. The correlation between two continuous variables was estimated by Pearson’s correlation. The comparison of glucose metabolism features of each cell cluster between different mice was conducted by one-way ANOVA. The comparison of FDG uptake in the human hippocampus was performed by Mann-Whitney tests with Bonferonni multiple test comparison. Correlation analysis between hippocampal FDG uptake and sTREM2 levels was performed by Spearman correlation analysis.

## Results

### FDG PET shows hippocampal hypermetabolism in AD mice

**Fig. 1:**
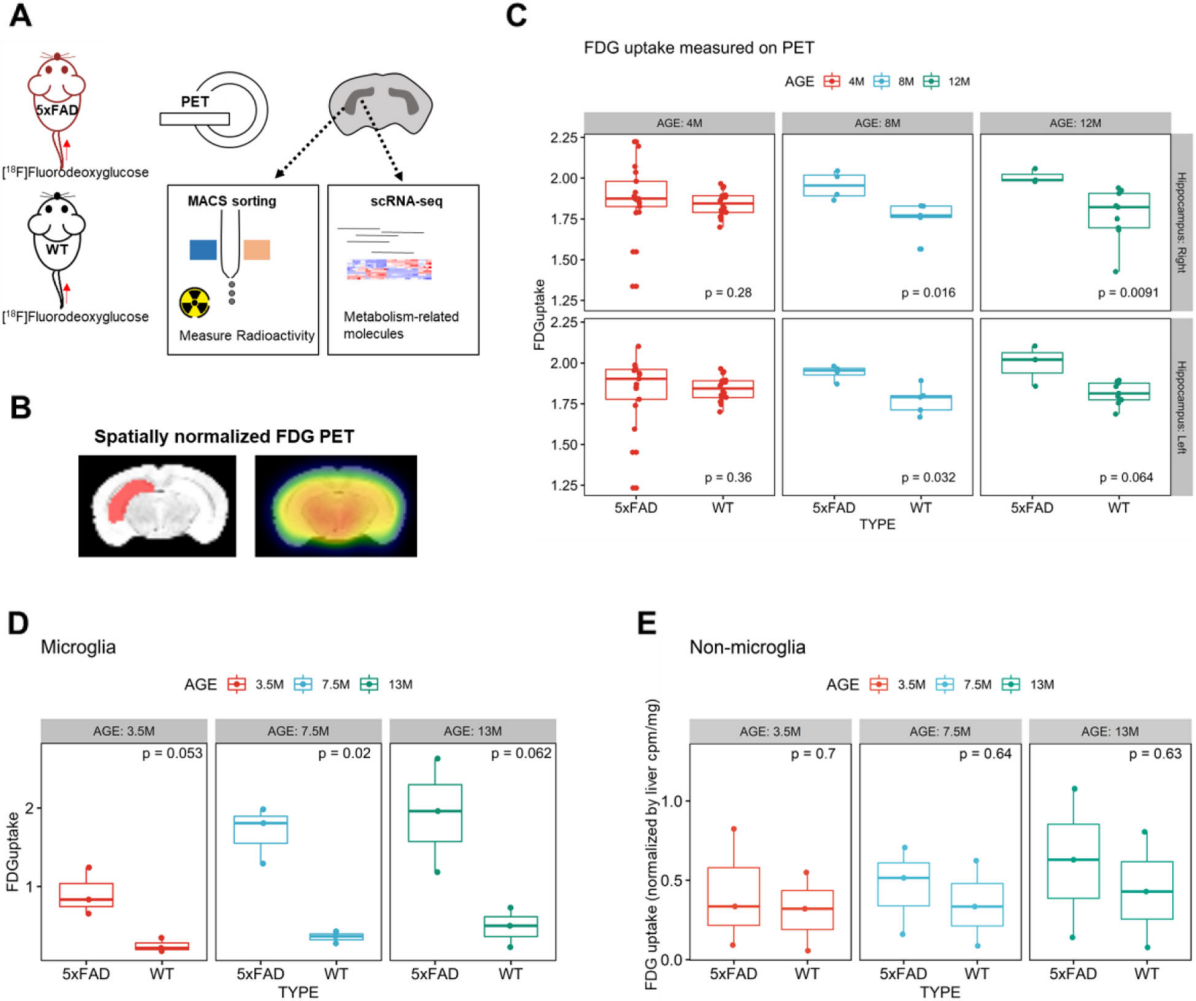
Increased hippocampal glucose uptake in Alzheimer’s disease mediated by microglia. **A** The overall workflow of the study using [^18^F]fluorodeoxyglucose (FDG) is represented. FDG was injected into 5xFAD mice of different ages and wild-type (WT) mice. Small-animal PET scans were acquired to non-invasively assess glucose uptake in the hippocampus. In addition, hippocampal cells were dissociated to analyze microglial FDG uptake as an *ex vivo* study. Single cell RNA-seq analyses were also performed by dissociating hippocampal cells. **B** To evaluate FDG uptake in the hippocampus on FDG PET, spatially normalized PET images and volume of interest for the hippocampus defined on the template MRI were used. **C** Four-month-old mice showed no significant difference in hippocampal glucose uptake of 5xFAD and WT (*p* = 0.28 and *p* = 0.36 for right and left hippocampus, respectively). Eight-month-old 5xFAD mice showed significantly higher FDG uptake in the hippocampus than WT (*p* = 0.016 and *p* = 0.032 for right and left hippocampus). Twelve-month-old 5xFAD mice also showed a similar pattern, with higher FDG uptake in the hippocampus than WT mice (*p* = 0.0091 and *p* = 0.064 for right and left hippocampus). **D** After FDG injection, the hippocampus was dissected, and microglia were dissociated. Ex vivo measurement of FDG activity showed that microglial uptake was significantly higher in 5xFAD at 7.5 months of age. Thirteen-month-old 5xFAD mice showed higher microglial FDG uptake than WT mice, although this difference did not reach statistical significance (*p* = 0.062). **E** Non-microglial cells of the hippocampus showed no significant difference in FDG uptake between 5xFAD and WT for all age groups

To investigate the metabolic change in the hippocampus of AD, we performed in vivo imaging using FDG PET and collected microglia to quantify FDG uptake. In addition, scRNA-seq of the hippocampus was also performed to investigate how cell-level metabolic gene signatures change in vivo (Fig. 1A). First, we compared FDG uptake in the hippocampus of 5xFAD mice and WT mice after preprocessing using a manually defined hippocampus on the normalized brain (Fig. 1B). As a result, 4-month-old mice showed no significant difference in hippocampal FDG uptake. Eight-month-old and 12-month-old 5xFAD mice showed significantly higher FDG uptake in the hippocampus than WT mice (*p* = 0.016 and *p* = 0.032, 8-month-old; *p* = 0.009 and *p* = 0.06, 12-month-old; right and left hippocampus, respectively) (Fig. 1C, Supplemental figure 1).

We then hypothesized that hippocampal hypermetabolism was associated with the accumulation of inflammatory cells. Ex vivo FDG uptake in microglia in the hippocampus was examined to reveal whether hippocampal hypermetabolism depended on metabolic changes in microglia or non-microglial cells. After FDG injection, microglia in the hippocampus were selectively collected to measure radioactivity. As a result, at 7.5 months of age, 5xFAD mice showed significantly higher FDG uptake in microglia than WT mice. At 3.5 and 13 months of age, 5xFAD mice also showed a trend of higher FDG uptake in microglia than WT mice. Other non-microglial cells showed no significant difference in FDG uptake regardless of age (Fig. 1D, E). We also acquired FDG uptake in subtypes of non-microglial cells, including astrocytes and neurons in 6-month-old 5xFAD. FDG uptake in microglia was higher in 5xFAD, while astrocytes and neurons showed no significant difference in FDG uptake between 5xFAD and WT mice (Supplementary figure 1B, C).

### Single-cell analysis reveals changes in glucose metabolic signatures of microglia of AD mice

**Fig. 2:**
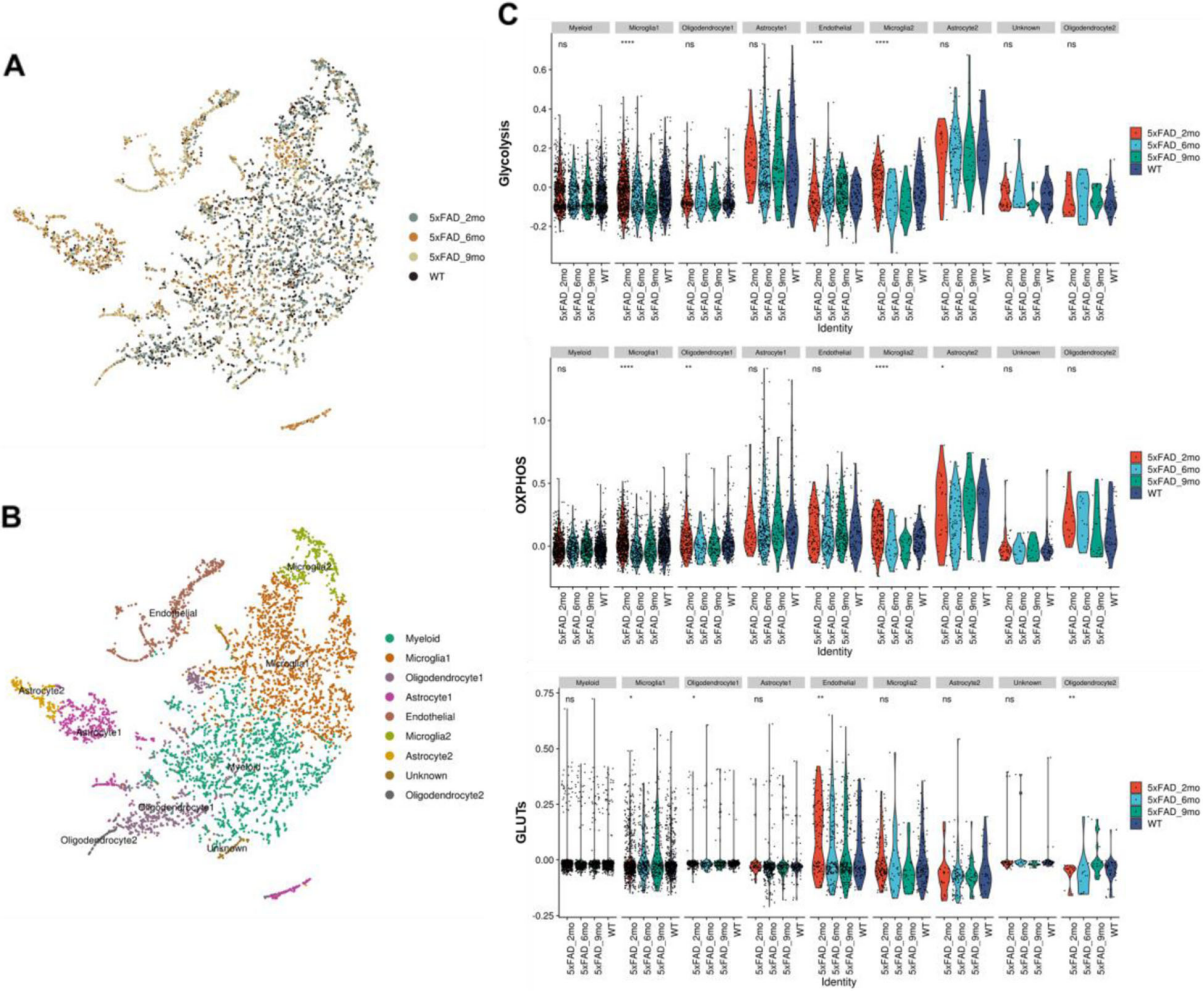
Single-cell level glucose metabolic features in the hippocampus of the AD mouse model. **A** RNA-sequencing data from a total of 5812 cells from 5xFAD mice (2-, 6-, and 9-month-old) and WT were analyzed. The t-distributed stochastic neighborhood embedding (t-SNE) map showed the different distribution of cells according to the progression of 5xFAD. **B** Each cell was clustered and named according to markers. **C** To analyze glucose metabolic features, the gene module was scored by gene sets of glycolysis, oxidative phosphorylation (OXPHOS), and glucose transporters (GLUTs). The enrichment score of each cell cluster derived from different groups was compared. Accordingly, a cluster, ‘microglia 1’ showed significantly different enrichment scores of glycolysis, OXPHOS, and GLUTs. Another microglia cluster, ‘microglia 2’, also showed significantly different enrichment scores of glycolysis and OXPHOS. (ns: not significant, *p < 0.05, **p < 0.01, ***p < 0.001, ****p < 0.0001)

As hippocampal hypermetabolism in 5xFAD was associated with increased uptake in the microglia of the hippocampus, we investigated the metabolic signatures of various cell types of the hippocampus at the single-cell level. The scRNA-seq data were obtained from the hippocampus of 5xFAD mice of different ages (2, 6, and 9 months old) and a WT mouse. A total of 5812 cells (1898, 1200, and 933 cells for 2-, 6-, and 9-month-old 5xFAD and 1781 cells for WT) were clustered and analyzed. Accordingly, nine different cell clusters were identified (Fig. 2A, B). The distribution of cell types was changed according to the aging of 5xFAD mice (Supplemental figure 2). The identified markers and alleged cell type-specific gene expression of each cluster are represented in Supplemental figure 3 and Supplemental figure 4. To compare the metabolic properties of each cell type, the enrichment scores of glycolysis, OXPHOS, and glucose transporters (GLUTs) were estimated. The enrichment scores of gene sets were evaluated for each cell. These glucose metabolism features were significantly different among 5xFAD mice of different ages and WT in specific clusters (Fig. 2C, Supplemental figure 5). In particular, glycolysis, OXPHOS, and GLUTs of different mice were significantly different in the microglia 1 cluster. For the microglia 1 cluster, the enrichment scores of glycolysis and OXPHOS were lower and the score of GLUTs was higher in the 9-month-old 5xFAD mouse. Another microglia cluster, the microglia 2 cluster, also showed different glycolysis and OXPHOS scores between different 5xFAD mice. The enrichment scores of glycolysis and OXPHOS of the microglia 2 cluster were lower in the 9-month-old 5xFAD mouse. Other clusters, including astrocytes and oligodendrocytes, did not show different glucose metabolic features among the different mice. Thus, we hypothesized that highly changed glucose metabolic signatures in microglia affected hippocampal glucose hypermetabolism.

### Reprogrammed glucose metabolism of microglial subtypes characterized by increased GLUT and decreased glycolysis and OXPHOS

**Fig. 3:**
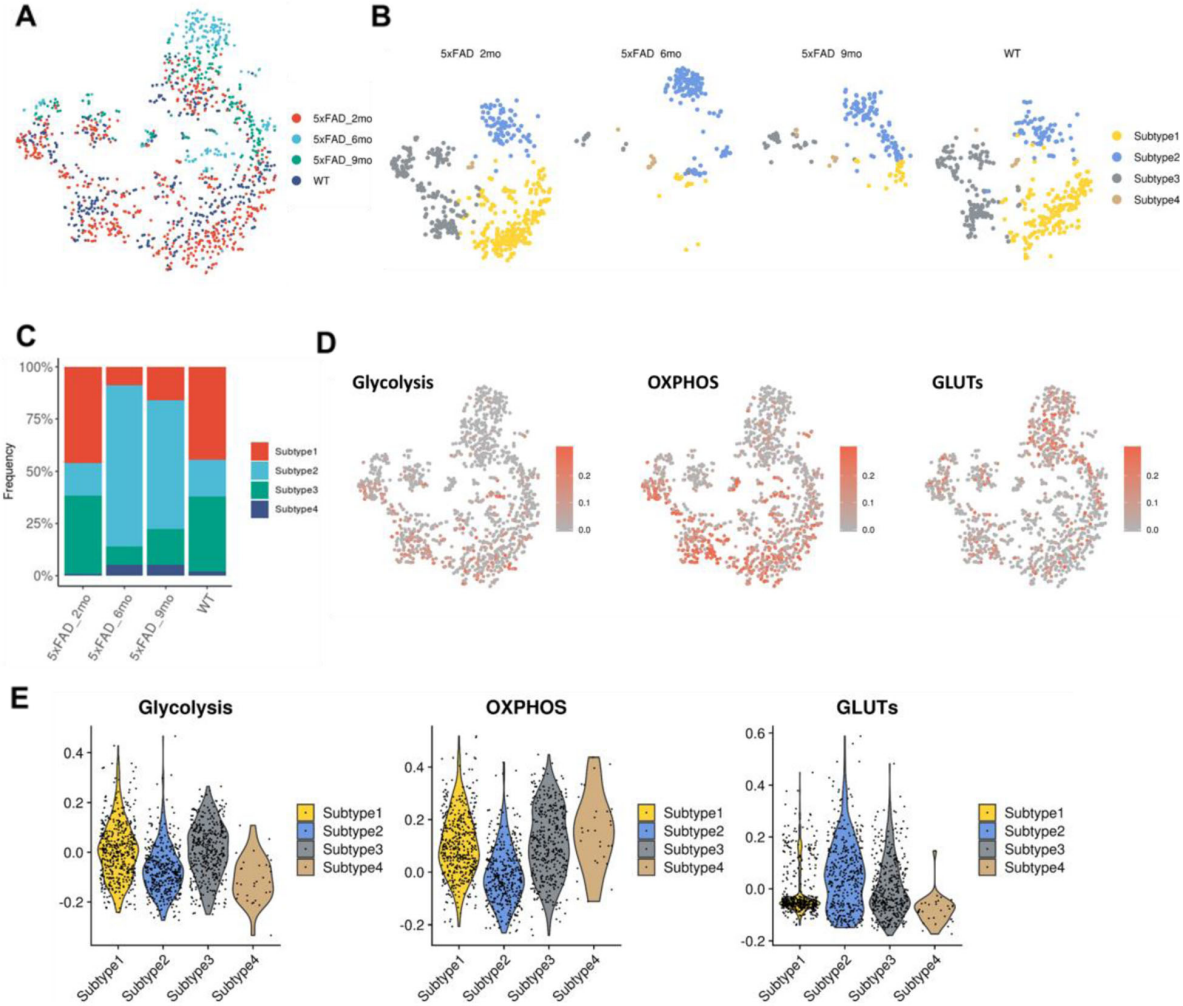
Reconstitution of glucose metabolism features of hippocampal microglia in the AD mouse model. **A** The t-SNE map represents the hippocampal microglia of different mouse groups. **B** Four different subtypes of hippocampal microglia were identified. The distribution pattern of microglial subtypes was changed according to mouse groups. **C** The proportion of subtypes of hippocampal microglia changed according to the age of 5xFAD mice. The 6-month-old and 9-month-old mice showed relatively more hippocampal microglia subtype 2. **D** The glucose metabolic features of microglial subtypes are represented. The enrichment score of GLUTs showed a different distribution pattern than glycolysis and OXPHOS enrichment scores. **E** Glucose metabolism features of the hippocampal microglial subtypes were different. Glycolysis and OXPHOS enrichment scores were lower in ‘Subtype 2’, while GLUT enrichment score was higher in ‘Subtype 2’ than other subtypes

As glucose metabolic features of microglia were significantly different between mice, microglial subsets were further investigated. Four different subtypes of microglia were identified (Fig. 3A). The markers of different microglial subtypes are represented in Supplemental figure 6. The proportion of subtypes of microglia changed according to the age of 5xFAD mice (Fig. 3B, C). In particular, subtype 1 and 3 were remarkably decreased in 6-month-old and 9-month-old 5xFAD mice. Subtype 2 was relatively increased in 6-month-old and 9-month-old 5xFAD mice.

The glucose metabolism features of microglial subtypes are represented (Fig. 3D). The enrichment scores of glycolysis and OXPHOS were relatively high in the subtype 1 and 3 microglia (Fig. 3E). On the other hand, subtype 2 microglia showed relatively low glycolysis and OXPHOS profiles and a high number of GLUTs. These findings showed that the progression of 5xFAD pathology was associated with the increased microglial subtypes with high GLUTs and decreased microglial subtypes with high OXPHOS and glycolysis.

### Hippocampal glucose metabolism as a surrogate of microglia-mediated inflammation in the human brain

**Fig. 4:**
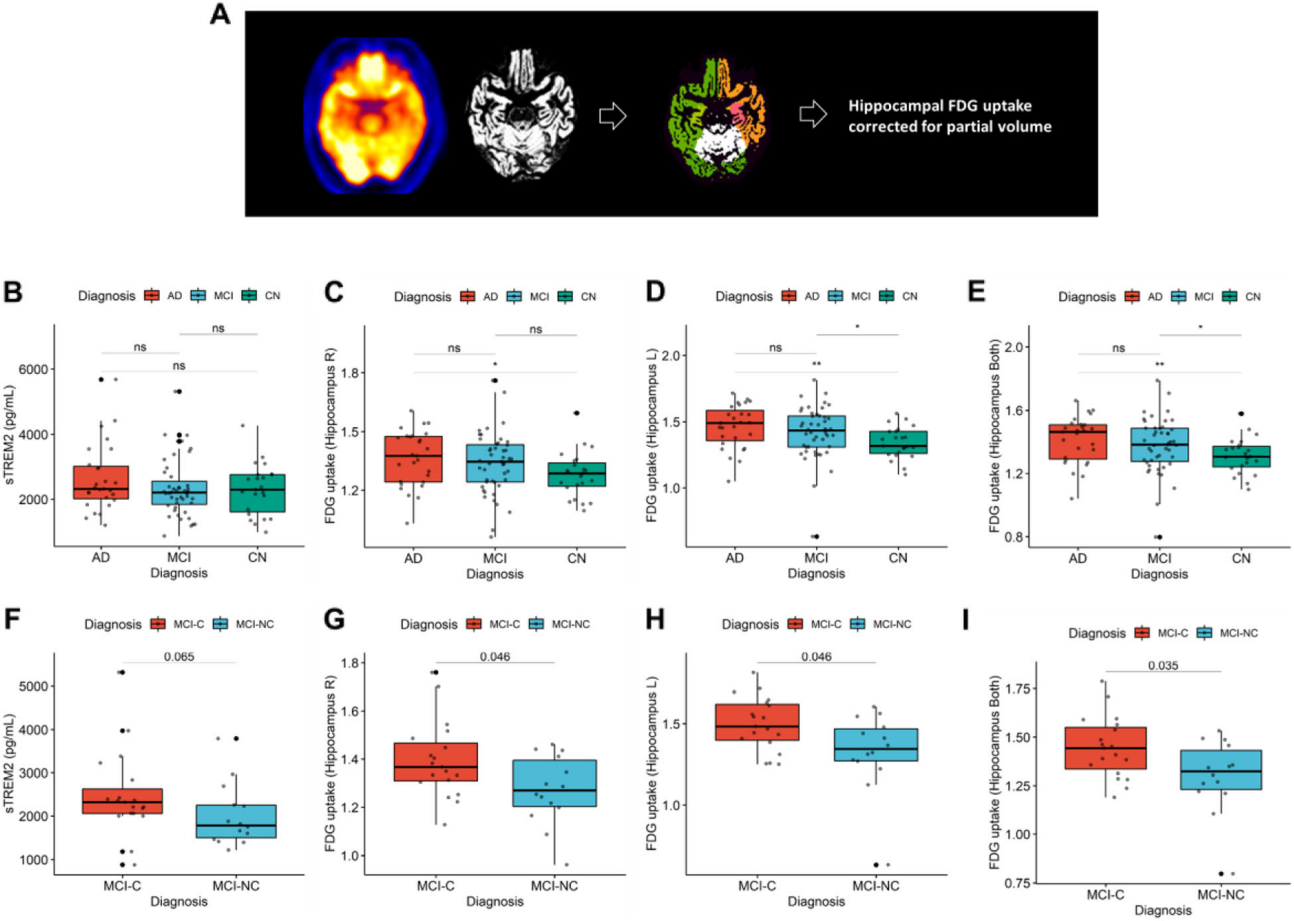
Hippocampal FDG uptake is higher in human AD and MCI. **A** As a human study, FDG uptake in the hippocampus was analyzed and compared with soluble TREM2 (sTREM2) obtained by CSF, a biomarker for evaluating microglia-mediated inflammation. FDG PET images and three-dimensional T1 MRI were coregistered with each other. Patient-wise volume of interest was automatically generated, and FDG uptake in the hippocampus was calculated with the correction of partial volume effect. **B** CSF sTREM2 of AD, MCI, and controls (CN) was not significantly different (2.63 ± 1.03 × 10^3^ vs. 2.35 ± 0.88 × 10^3^ vs. 2.30 ± 0.79 × 10^3^; *p* = 0.45). However, the hippocampal FDG uptake of different groups was different. **C** For the right hippocampus, FDG uptake of different groups, AD, MCI, and CN, showed a marginal difference. AD showed marginally higher FDG uptake in the right hippocampus than CN (1.36 ± 0.14 vs. 1.34 ± 0.16 vs. 1.28 ± 0.12; *p* = 0.03). **D** FDG uptake in the left hippocampus was significantly different between the groups (1.46 ± 0.17 vs. 1.42 ± 0.20 vs. 1.34 ± 0.12; *p* = 0.02). AD and MCI showed higher FDG uptake in the left hippocampus compared with CN (*p* = 0.007 for AD vs. CN; *p* = 0.02 for MCI vs. CN). **E** We also measured FDG uptake using a volume-of-interest including both hippocampi. FDG uptake in both hippocampi was also significantly different between the groups (1.41 ± 0.15 vs. 1.38 ± 0.17 vs. 1.31 ± 0.11; *p* = 0.023). AD and MCI showed higher FDG uptake in both hippocampi compared with CN (*p* = 0.008 for AD vs CN; *p* = 0.04 for MCI vs CN). **F** CSF sTREM2 of MCI-C was higher than that of MCI-NC, although it showed borderline significance (2.49 ± 0.98 × 10^3^ vs. 2.13 ± 0.72 × 10^3^; *p* = 0.065). **G** For the right hippocampus, FDG uptake of MCI-C and MCI-NC was significantly different (1.39 ± 0.16 vs. 1.29 ± 0.14; *p* = 0.046). **H** For the left hippocampus, FDG uptake of MCI-C and MCI-NC was also significantly different (1.50 ± 0.16 vs. 1.35 ± 0.22; *p* = 0.046). **I** For both hippocampi, FDG uptake of MCI-C and MCI-NC was also significantly different (1.45 ± 0.16 vs. 1.32 ± 0.18; *p* = 0.035)

Since the mouse FDG PET study and scRNA-seq analyses suggested that microglial reconfiguration underlies hippocampal hypermetabolism, we explored whether the hippocampal metabolism evaluated in human imaging could be a surrogate of metabolic changes in microglia as an AD pathology (Fig. 4A). We used the human data of sTREM2 measured in CSF as surrogates of microglial inflammation. The sTREM2 level was not different among the three diagnostic groups, AD, MCI, and CN (Fig. 4B). The hippocampal FDG uptake was estimated by using a partial volume correction method based on structural MR images [35]. Accordingly, hippocampal FDG uptake was significantly increased in the AD brain compared with the CN brain (Fig. 4C–E). We compared hippocampal FDG uptake of patients with MCI-C and MCI-NC to investigate whether it could be a predictive marker for the progression of MCI. The sTREM2 of MCI-C was higher than that of MCI-NC, although it showed borderline statistical significance (Fig. 4F). FDG uptake was significantly higher in MCI-C than MCI-NC in right and left hippocampus (Fig. 4G–I).

**Fig. 5:**
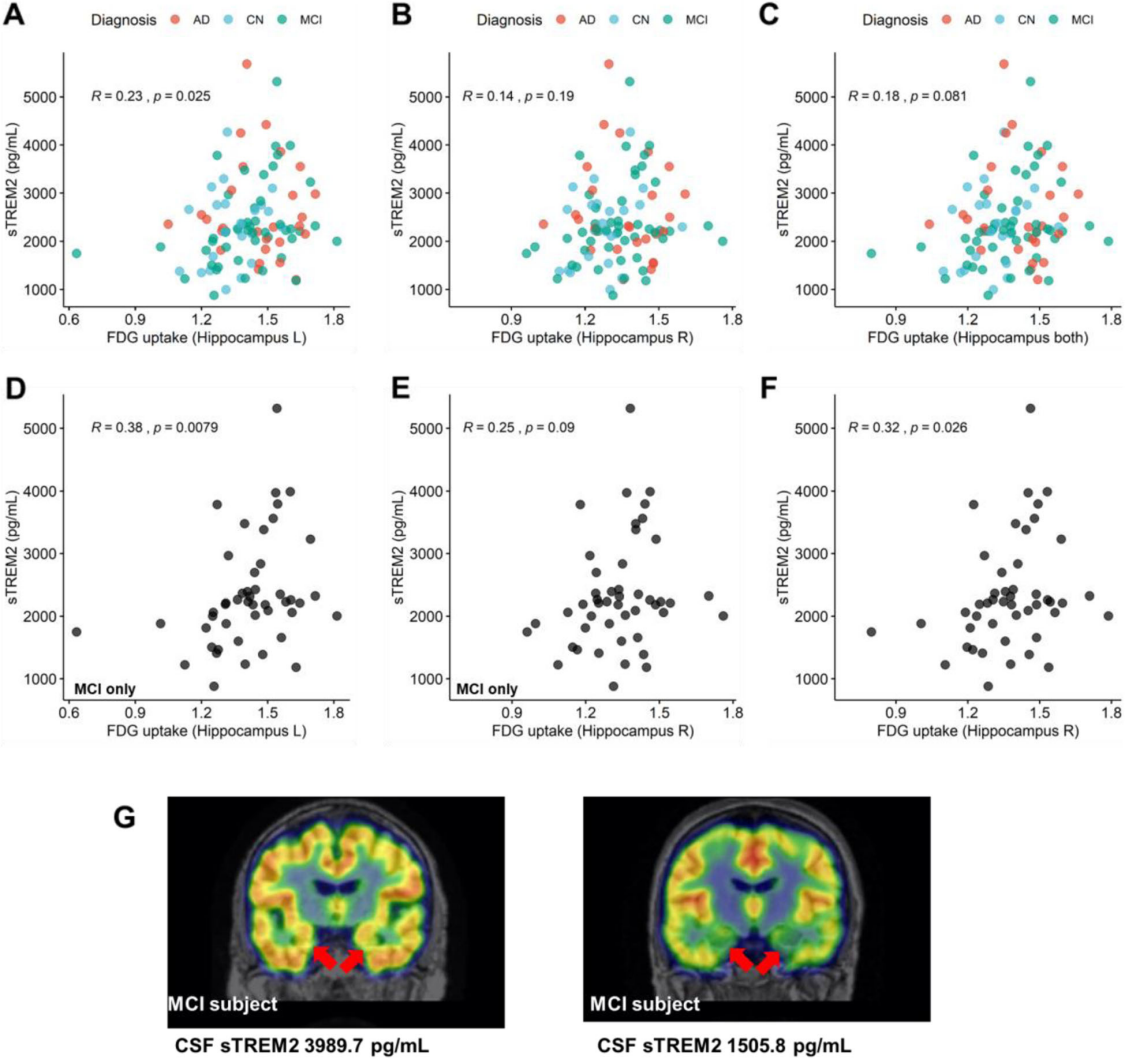
Hippocampal FDG uptake and CSF biomarkers for microglia-mediated inflammation. Hippocampal FDG uptake showed a trend of positive correlation with CSF sTREM2. **A** The left hippocampal FDG uptake showed a significant positive correlation with sTREM2 (*r* = 0.23, *p* = 0.03). **B** The right hippocampal FDG uptake showed a weak positive correlation with CSF sTREM2 (*r* = 0.14, *p* = 0.19), which did not reach statistical significance. **C** Hippocampal FDG uptake in both hippocampi also showed a weak positive correlation with CSF sTREM2 (*r* = 0.18, *p* = 0.08). **D**–**F** A subgroup, MCI showed a positive correlation between sTREM2 and hippocampal FDG uptake (*r* = 0.38, *p* = 0.008 for the left hippocampus; *r* = 0.25, *p* = 0.09 for the right hippocampus; *r* = 0.32, *p* = 0.026 for both hippocampi). **G** Two representative cases of MCI patients who showed different FDG uptake patterns. An MCI patient with relatively high CSF sTREM2 had increased FDG uptake in the medial temporal lobes, while another MCI patient with relatively low CSF sTREM2 had a hypometabolic pattern in the medial temporal lobe

sTREM2 showed a trend of positive correlation with hippocampal FDG uptake (*r* = 0.23, *p* = 0.025 for left hippocampal FDG uptake; *r* = 0.14, *p* = 0.19 for right hippocampal FDG uptake; *r* = 0.18, *p* = 0.081 for both hippocampi; Fig. 5A–C). These positive associations were more prominent in the subgroup of MCI subjects (Fig. 5D–F). The association between sTREM2 and hippocampal FDG uptake was not found in AD and CN (Supplemental figure 7). However, hippocampal FDG uptake of AD, MCI, and CN was not associated with the severity of a cognitive score, Alzheimer’s disease assessment scale (ADAS13) (Supplementary figure 8). sTREM2 was also not correlated with ADAS13 in all subgroups (AD, MCI and CN). The representative cases of FDG PET images of MCI subjects with different sTREM2 levels are represented in Fig. 5E. Relatively high FDG uptake in medial temporal cortices was found in a subject with higher sTREM2 levels.

## Discussion

The evaluation of neuroinflammation in AD become an important pathophysiologic biomarker as it has been demonstrated to be a predictor of cognitive decline in patients using CSF inflammation markers as well as animal models [36, 37]. As a key player in neuroinflammation, it is necessary to understand the characteristics of microglia in AD brain and observe changes of microglial activation to predict progression and cognitive changes in AD continuum.

One of the key changes in microglia-mediated inflammation is metabolism, as immune cells depend on various metabolic pathways according to energy demands. The activation of the innate immune system is associated with a metabolic shift from oxidative phosphorylation (OXPHOS) to glycolysis, which is a rapid process of energy production [8]. These active metabolic processes can be suppressed by chronic inflammatory processes due to broad defects in energy metabolism that underlie immunoparalysis [13]. This close link between metabolism and immune cells is also found in AD. TREM2, a key surface receptor for the microglial response to neurodegeneration-related proteins, was associated with the metabolic fitness of microglia to enhance glycolysis to protect them from neurodegenerative proteins. According to a previous study, TREM2-deficiency induces impaired energy metabolism in microglia associated with defective mTOR pathway [14]. Furthermore, a recent study showed that amyloid beta exposure to microglia induced microglial reprogramming from OXPHOS to glycolysis at the acute phase; however, both were reduced after chronic exposure similar to the process of immunoparalysis [15]. As microglia with pro-inflammatory response, so-called M1phenotypes in macrophages, are associated with increased glycolysis [38], this energy defect implies immune tolerance mediated by AD pathology in the hippocampus. Notably, while both glycolysis and OXPHOS were decreased, glucose uptake of hippocampal microglia was increased in our study. This increased glucose uptake was identified in ex vivo microglia as well as in vivo PET imaging. Moreover, a subset of microglia with high GLUTs was increased in 5xFAD mice. As increased GLUT1 is similar with the common pathway of stress condition even in broad defects of energy metabolism [39], the finding of increased GLUT1 and FDG uptake in hippocampal microglia could be associated with metabolic reprogramming of chronic inflammatory changes resulting from AD pathology. The specific metabolic pathways of microglia with immunoparalysis are unknown, and possibly, increased storage pathways during both decreased glycolysis and OXPHOS. As future work, metabolic pathways according to microglial activation status should be studied to fully understand the metabolic pathway of glucose of microglia with increased GLUT1 with immunoparalysis. Furthermore, scRNA-seq data were acquired from limited mice as well as limited brain regions. Considering the spatial diversity in microglia [40] and different metabolic patterns of hippocampus on PET according to our study, further detailed scRNA-seq analyses should be performed to understand glucose metabolism of various immune cell types in the brain.

Recent progress has been made in image-based phenotyping of AD, and the changes in key biomarkers are spatially and temporally different according to disease progression [41, 42]. For example, volume loss evaluated by MRI is typically found in the hippocampus, while reduced glucose metabolism is mainly found in the posterior cingulate, lateral temporal, superior parietal, and medial frontal cortices [42]. The reduced glucose metabolism was spatially associated with amyloid deposits and regarded as decreased neuronal activity resulting from AD pathology [43]. While the precuneus and posterior cingulate cortex are the common brain regions that show both hypometabolism and volume loss according to the progression of AD [41], FDG uptake patterns in the hippocampus are equivocal. Despite the consistent association of hippocampal atrophy and cognitive decline, FDG uptake in the hippocampus is relatively preserved and sometimes increased [22, 23, 44]. The animal AD model has also showed equivocal results. A recent study presented regional FDG uptake in 12-month-old 5XFAD mice using autoradiography as well as PET, while it showed no definite difference in FDG uptake compared with wild types [45]. In spite of different protocols and experimental settings from our animal PET studies, this study showed insignificantly higher FDG uptake in the female 5XFAD mice hippocampus than female wild-type mice [45]. The results of increased FDG uptake among these studies were associated with partial volume correction as hippocampal atrophy could cause underestimate the measurement of FDG uptake on PET. According to our findings, one of the reasons for relatively preserved glucose uptake was microglial reconfiguration in the hippocampus. Furthermore, the microglia-mediated inflammatory response alters adult hippocampal neurogenesis [46], which may also affect FDG uptake in the hippocampus. Notably, as FDG uptake usually depends on neuronal activity, hippocampal FDG uptake patterns on PET could depend on multiple cell types. Accordingly, glucose uptake in the hippocampus non-invasively measured by FDG PET could be a biomarker associated with microglia-mediated inflammation. Notably, microglia-mediated inflammation is increased at the early stage of AD and reduced at the late stage according to CSF inflammatory markers [47, 48]. In this regard, FDG uptake in the hippocampus reflecting microglial reconstitution could be varied in AD, increased at the early stage and reduced at the late stage. Further study is needed to assess hippocampal FDG uptake according to disease progression.

Recently, the role of microglia in AD pathophysiology has received considerable attention. In particular, DAM were identified in neurodegenerative disorders and regarded as a potential target of treatment [49]. The key hypothesis is the protective role of DAM in the immune response to neural tissue damage, such as innate immune response to damage-associated molecular patterns [5]. Thus, in terms of clinically applicable biomarker development, a subset of microglia that reflects the current status of neuroinflammation caused by AD pathophysiology is needed because microglia are highly heterogeneous and dynamically changed. To date, TSPO PET has been widely studied in AD to non-invasively evaluate microglial inflammation. However, TSPO-positive microglia are different from DAM activated by the TREM2 pathway [50, 51]. In this regard, it is difficult to reflect complex and dynamic changes in microglial subsets in neurodegenerative disorders. Another emerging biomarker for assessing DAM is CSF sTREM2, which can induce DAM to protect against amyloid beta-induced neuronal damage [52, 53]. According to previous studies, sTREM2 can reflect disease progression in terms of microglia-mediated inflammation and be a potential biomarker of AD [54]. Despite the emerging role of CSF sTREM2 in reflecting microglia-mediated inflammation, one of the limitations considering clinical usage is its invasiveness. As we found that sTREM2 in CSF was positively correlated with hippocampal glucose uptake non-invasively measured by FDG PET, it could be a clinically feasible surrogate to assess microglia-mediated inflammation. Furthermore, FDG PET is a common and widely used imaging modality for study of AD; thus, it is relatively easy to use as a surrogate biomarker. Interestingly, sTREM2 was particularly correlated with hippocampal FDG uptake in MCI patients among whom converters showed higher uptake than non-converters. Furthermore, the correlation between hippocampal FDG uptake and sTREM2 was mainly found in MCI, while it was not correlated in AD and CN. It supports microglia-mediated inflammation during the disease course as sTREM2 is elevated at the early stage of AD and decreased at the late stage of AD [47, 55]. Hippocampal FDG uptake measured by PET in late stage AD or normal aged subjects may mainly depend on non-microglial cells due to relatively low glucose uptake in microglia. This might be a cause of the lack of correlation between sTREM2 and hippocampal FDG uptake in AD patients and normal controls. Thus, it does not reflect microglial metabolic status related to the sTREM2 pathway for normal subjects or late stage AD. Therefore, hippocampal FDG uptake, as well as sTREM2, could be a good biomarker of MCI or early AD for predicting outcome. Additional validation studies for investigating FDG uptake in the hippocampus according to the course of AD and the microglial activation pathway will test this hypothesis.

## Conclusions

We found that increased glucose uptake in the hippocampus was associated with microglial reconfiguration. At the single-cell level, a subset of hippocampal microglia that showed high GLUTs and low glycolysis and OXPHOS was associated with the progression of the AD mouse model. This microglia-mediated glucose uptake in the hippocampus could be estimated by FDG PET, one of the most widely used non-invasive imaging modality. This association between microglia-mediated inflammation and FDG uptake in the hippocampus was recapitulated in human imaging by the positive correlation of CSF sTREM2 and hippocampal FDG uptake. Microglia-mediated inflammation in the brain has been regarded as a key pathophysiology of AD. Nonetheless, because of complicated dynamic changes in microglia affecting both the protection and progression of AD pathology represented by amyloid beta, clinical translation of these biological changes of dysfunctional microglia has been limited. Our findings will provide a clinically feasible surrogate marker to monitor the status of microglia-mediated inflammation by using widely used PET imaging. Furthermore, the characteristic microglial metabolism of AD analyzed by integrating imaging, scRNA-seq, and human biomarkers provides insight into new therapeutic targets of AD pathophysiology.

## Supplementary Information

**Additional file 1: Supplemental figure 1**. FDG PET images of mice with different ages and cellular FDG uptake. (A) FDG PET images averaged across each group were represented. Note that the hippocampal FDG uptake was estimated by pre-defined volume-of-interests after the spatial normalization of FDG PET. The hippocampal FDG uptake was increased in 8-month-old and 12-month-old 5xFAD mice compared with wild type mice with same age. (B) To know FDG uptake difference in subtypes of nonmicroglial cells, we additionally performed FDG uptake tests after cellular sorting including astrocytes and neurons in 6-month-old 5xFAD mice and WT. FDG uptake of astrocytes and neurons was not significantly different between 5xFAD and WT (*: *p* < 0.05). (C) Purity of cell fractions for ex vivo FDG studies was determined by PCR. The microglia-rich fraction was identified as *Hexb*, the astrocyte-rich fraction as *Slc1a3*, and the neuron-rich fraction as *Snap25*. Cortical lysates were used as positive controls. **Supplemental figure 2**. The change in cellular landscape of hippocampus according to aging of 5xFAD mice. t-SNE maps representing single cell-level transcripts were drawn for 5xFAD and wild type mice with different ages. **Supplemental figure 3**. Markers of hippocampal cell cluster. Cells were clustered according to the single cell-level transcripts and markers were identified. 9 different cell types were clustered and names were defined by alleged markers. The average expression value of cells of each cluster was represented. **Supplemental figure 4**. Expression of cell-type specific genes. Gene expression levels of cell-type specific genes were represented with t-SNE maps. **Supplemental figure 5**. The enrichment score of glucose metabolism-related molecules for hippocampal cells. t-SNE maps were drawn with the enrichment score of glucose metabolism modules. The enrichment score of glycolysis, oxidative phosphorylation (OXPHOS), and glucose transporters (GLUTs) was estimated by using KEGG pathway. The enrichment score was represented according to colormaps. Notably, astrocyte shows relatively higher glycolysis. GLUTs were highly expressed in endothelial cells and some types of microglial clusters. **Supplemental figure 6**. Markers of microglial subtypes. Microglial subsets were further analyzed to investigate reprogrammed microglia according to the AD progression particularly in terms of the glucose metabolic profiles. Microglia were clustered into 4 subtypes by gene expression data and markers were identified. **Supplemental figure 7**. The correlation between hippocampal FDG uptake and CSF sTREM2 in AD and controls. The hippocampal FDG uptake was positively correlated with CSF sTREM2 particularly in MCI patients, while the association was neither found in AD nor controls. FDG uptake in the left hippocampus (A) and the right hippocampus (B) was not significantly correlated with sTREM2 for AD patients, while the correlation was negative. Additionally, FDG uptake in the left hippocampus (C) and the right hippocampus (D) was not significantly correlated sTREM2 for controls. However, the correlation was positive though it did not reach a statistical significance. **Supplemental figure 8**. The correlation between the cognitive score and hippocampal FDG uptake. In the subgroups according to the diagnosis (CN, MCI and AD), hippocampal FDG uptake was not associated with the severity of a cognitive score, Alzheimer's disease assessment scale (ADAS13) (*above*). In addition, sTREM2 was also not correlated with ADAS13 in all subgroup (AD, MCI and CN) (*below*).

## Data Availability

Single cell RNA-sequencing data are available at Gene Expression Omnibus (GSE150934). Human brain imaging data and clinical features are available in ADNI database (http://adni.loni.usc.edu/).

## Abbreviations

AD: Alzheimer’s disease
DAM: Disease-associated microglia
PET: Positron emission tomography
TSPO: Translocator protein-18 kDa
FDG: Fluorodeoxyglucose
WT: Wild-type
scRNA-seq: Single-cell RNA-sequencing
OSEM: Ordered-subsets expectation maximum
MACS: Magnetic-activated cell sorting
CCA: Canonical correlation analysis
t-SNE: t-distributed stochastic neighborhood embedding
PCA: Principal component analysis
KEGG: Kyoto encyclopedia of genes and genomes
sTREM2: Soluble TREM2
ADNI: Alzheimer disease neuroimaging initiative
CN: Normal controls
MCI: Mild cognitive impairment
MCI-C: MCI converters
MCI-NC: MCI non-converters
OXPHOS: Oxidative phosphorylation
GLUT: Glucose transporter
