# Supplementary material for: Hippocampal glucose uptake as a surrogate of metabolic change of microglia in Alzheimer’s disease
Source: J Neuroinflammation. 2021 Aug 31;18:190. doi: 10.1186/s12974-021-02244-6 (PMC8408933; doi:10.1186/s12974-021-02244-6)
Supplement: Supplementary file 1 — Additional file 1: Supplemental figure 1. FDG PET images of mice with different ages and cellular FDG uptake. (A) FDG PET images averaged across each group were represented. Note that the hippocampal FDG uptake was estimated by pre-defined volume-of-interests after the spatial normalization of FDG PET. The hippocampal FDG uptake was increased in 8-month-old and 12-month-old 5xFAD mice compared with wild type mice with same age. (B) To know FDG uptake difference in subtypes of nonmicroglial cells, we additionally performed FDG uptake tests after cellular sorting including astrocytes and neurons in 6-month-old 5xFAD mice and WT. FDG uptake of astrocytes and neurons was not significantly different between 5xFAD and WT (*: p < 0.05). (C) Purity of cell fractions for ex vivo FDG studies was determined by PCR. The microglia-rich fraction was identified as Hexb, the astrocyte-rich fraction as Slc1a3, and the neuron-rich fraction as Snap25. Cortical lysates were used as positive controls. Supplemental figure 2. The change in cellular landscape of hippocampus according to aging of 5xFAD mice. t-SNE maps representing single cell-level transcripts were drawn for 5xFAD and wild type mice with different ages. Supplemental figure 3. Markers of hippocampal cell cluster. Cells were clustered according to the single cell-level transcripts and markers were identified. 9 different cell types were clustered and names were defined by alleged markers. The average expression value of cells of each cluster was represented. Supplemental figure 4. Expression of cell-type specific genes. Gene expression levels of cell-type specific genes were represented with t-SNE maps. Supplemental figure 5. The enrichment score of glucose metabolism-related molecules for hippocampal cells. t-SNE maps were drawn with the enrichment score of glucose metabolism modules. The enrichment score of glycolysis, oxidative phosphorylation (OXPHOS), and glucose transporters (GLUTs) was estimated by using KEGG pathway. [file 12974_2021_2244_MOESM1_ESM.pdf]

Hippocampal glucose uptake as a surrogate of metabolic change of microglia in Alzheimer's disease

Supplemental Figures

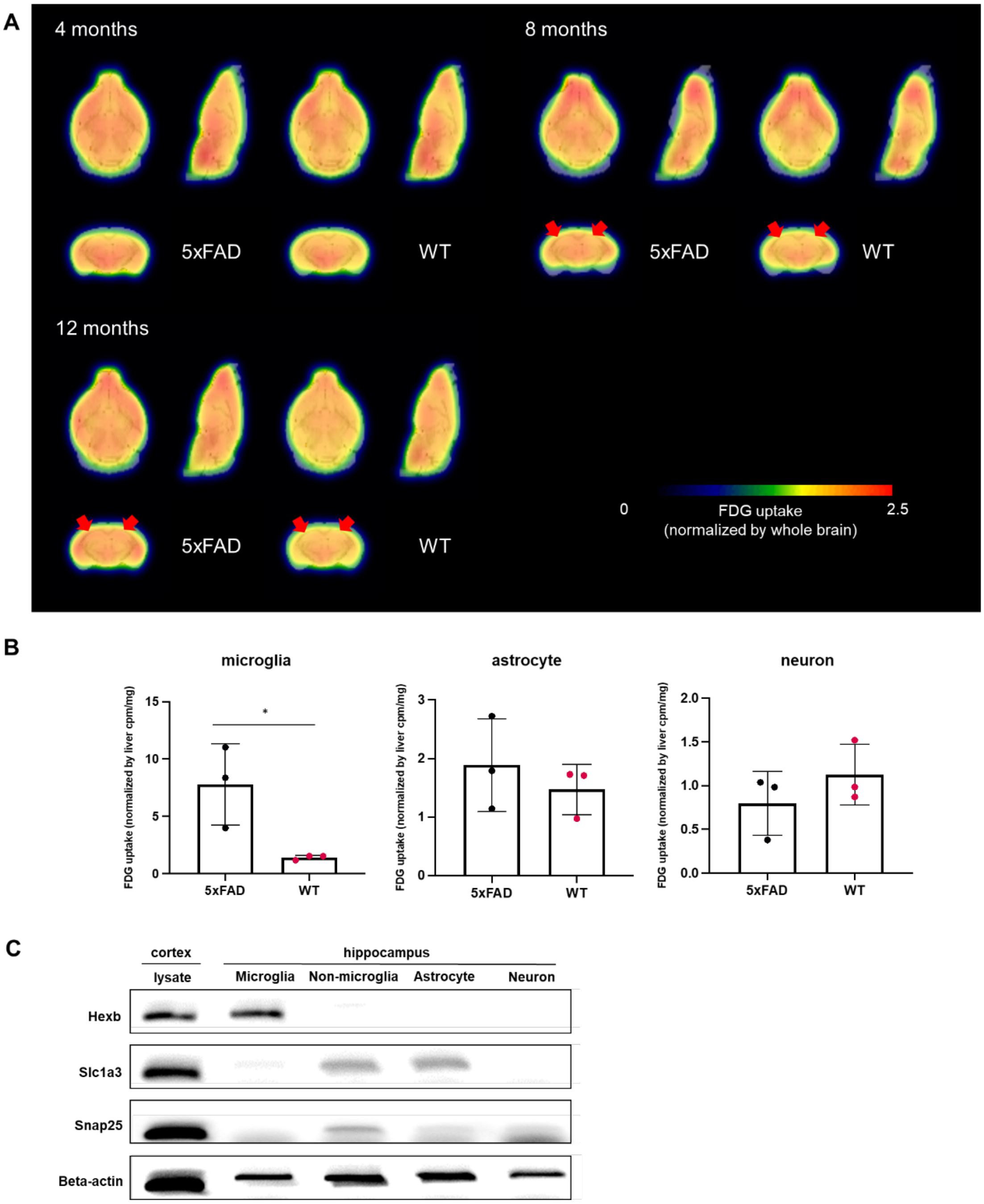

Supplemental figure 1. FDG PET images of mice with different ages and cellular FDG uptake. (A) FDG PET images averaged across each group were represented. Note that the hippocampal FDG uptake was estimated by pre-defined volume-of-interests after the spatial normalization of FDG PET. The hippocampal FDG uptake was increased in 8-month-old and 12-month-old 5xFAD mice compared with wild type mice with same age. (B) To know FDG uptake difference in subtypes of nonmicroglial cells, we additionally performed FDG uptake tests after cellular sorting including astrocytes and neurons in 6-month-old 5xFAD mice and WT. FDG uptake of

astrocytes and neurons was not significantly different between 5xFAD and WT (*: $p < 0.05$). (C) Purity of cell fractions for ex vivo FDG studies was determined by PCR. The microglia-rich fraction was identified as *Hexb*, the astrocyte-rich fraction as *Slc1a3*, and the neuron-rich fraction as *Snap25*. Cortical lysates were used as positive controls.

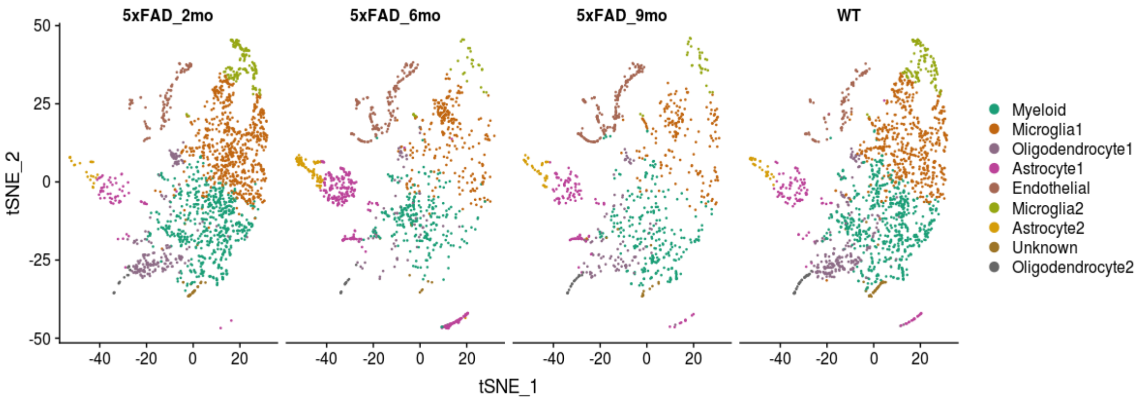

Supplemental figure 2. The change in cellular landscape of hippocampus according to aging of 5xFAD mice. t-SNE maps representing single cell-level transcripts were drawn for 5xFAD and wild type mice with different ages.

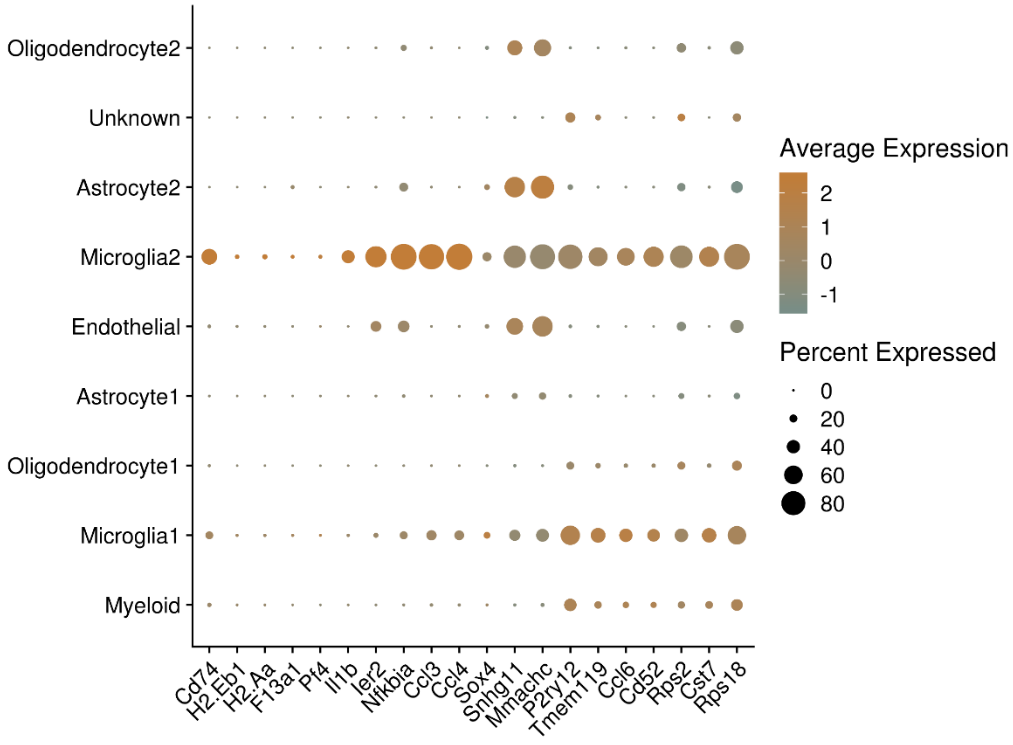

Supplemental figure 3. Markers of hippocampal cell cluster. Cells were clustered according to the single cell-level transcripts and markers were identified. 9 different cell types were clustered and names were defined by alleged markers. The average expression value of cells of each cluster was represented.

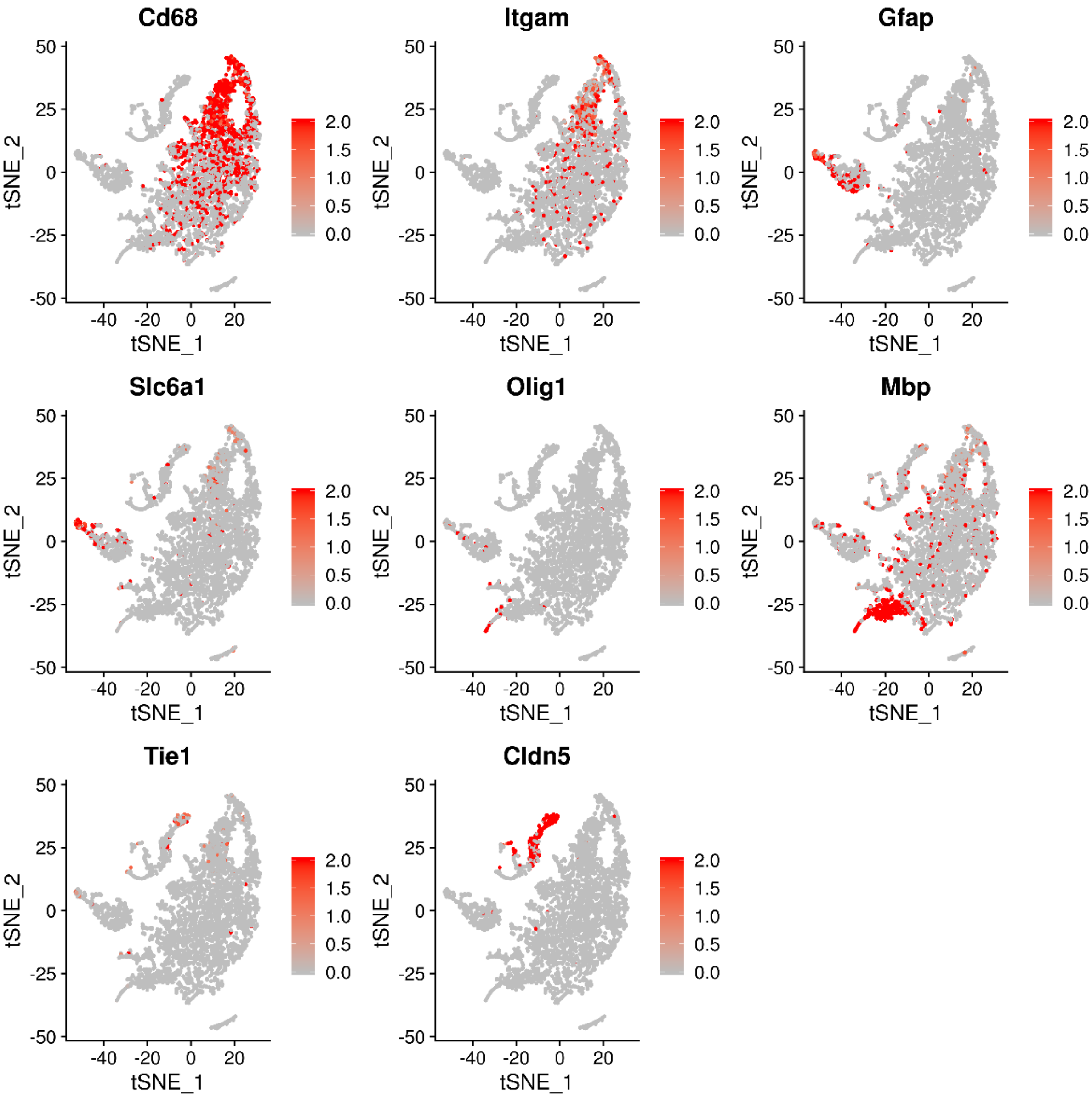

Supplemental figure 4. Expression of cell-type specific genes. Gene expression levels of cell-type specific genes were represented with t-SNE maps.

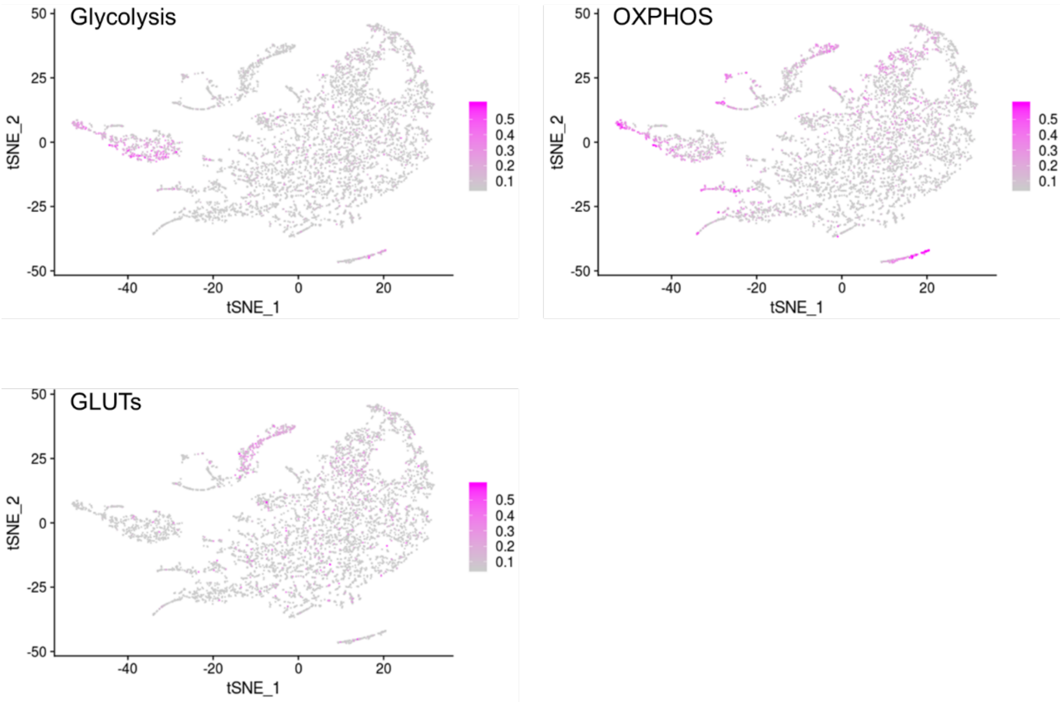

Supplemental figure 5. The enrichment score of glucose metabolism-related molecules for hippocampal cells. t-SNE maps were drawn with the enrichment score of glucose metabolism modules. The enrichment score of glycolysis, oxidative phosphorylation (OXPHOS), and glucose transporters (GLUTs) was estimated by using KEGG pathway. The enrichment score was represented according to colormaps. Notably, astrocyte shows relatively higher glycolysis. GLUTs were highly expressed in endothelial cells and some types of microglial clusters.

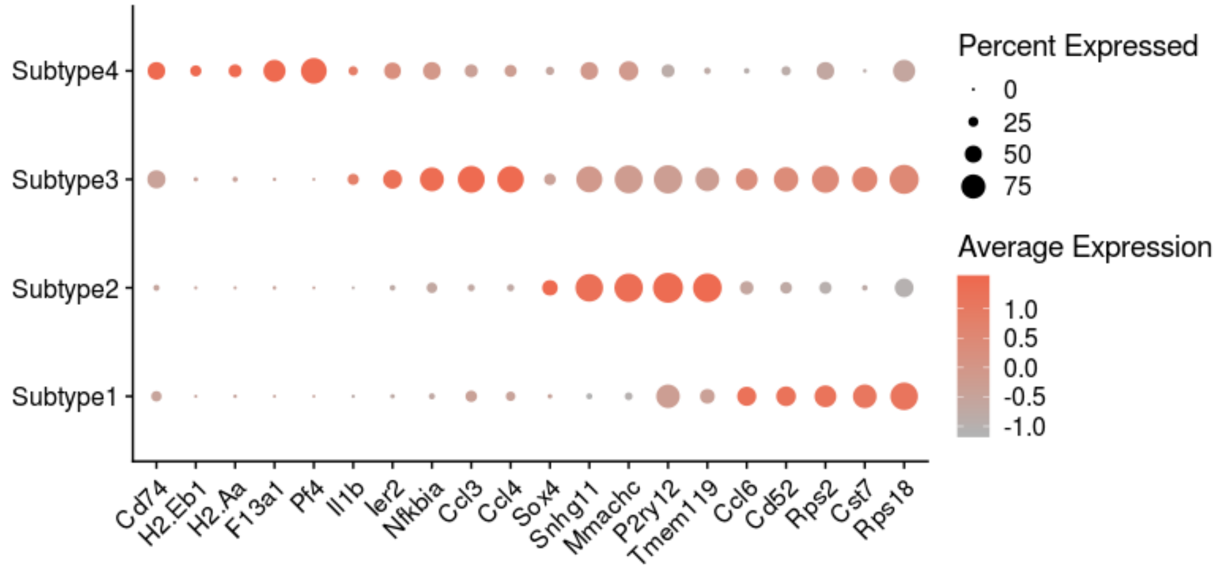

Supplemental figure 6. Markers of microglial subtypes. Microglial subsets were further analyzed to investigate reprogrammed microglia according to the AD progression particularly in terms of the glucose metabolic profiles. Microglia were clustered into 4 subtypes by gene expression data and markers were identified.

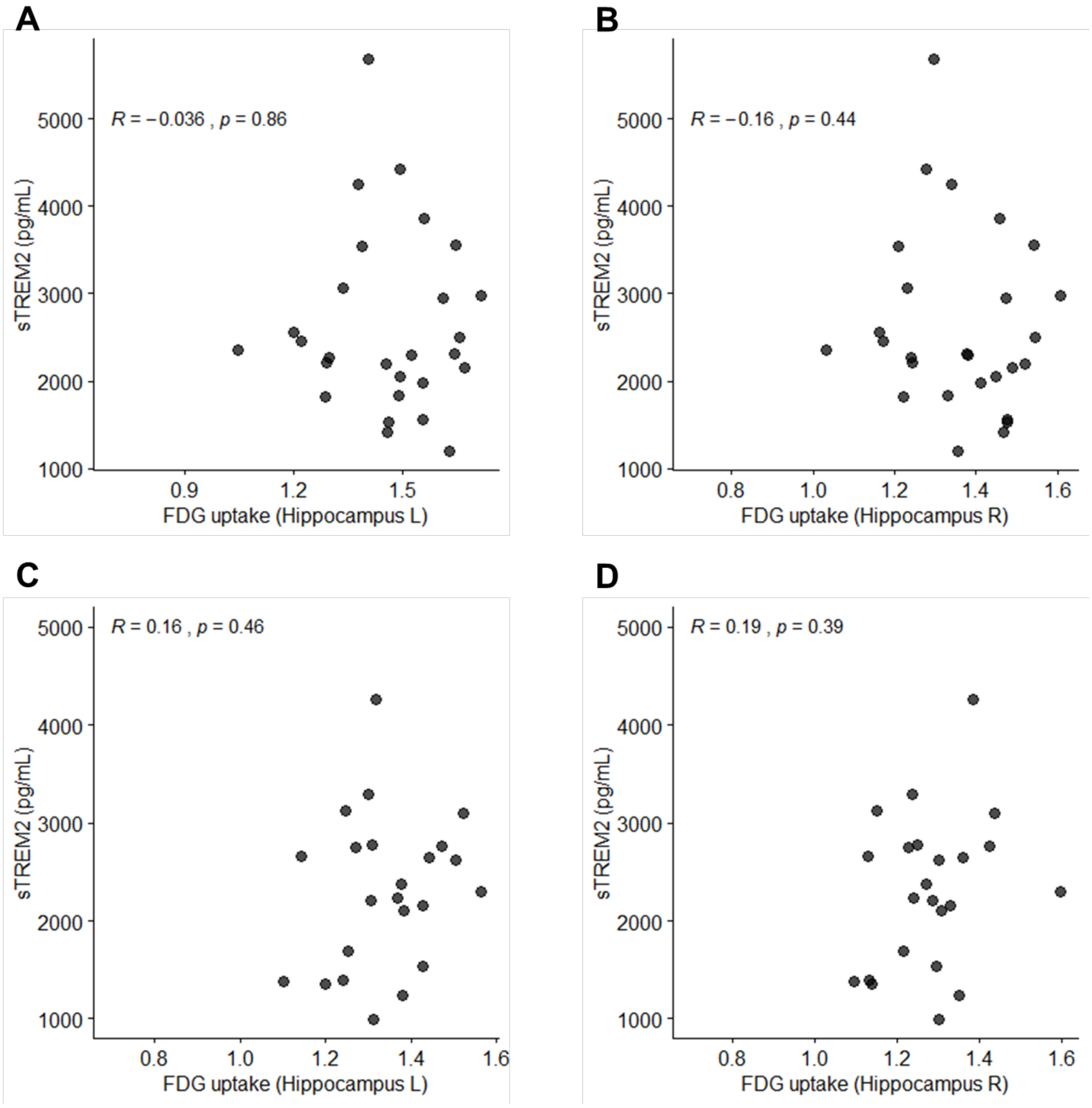

Supplemental figure 7. The correlation between hippocampal FDG uptake and CSF sTREM2 in AD and controls. The hippocampal FDG uptake was positively correlated with CSF sTREM2 particularly in MCI patients, while the association was neither found in AD nor controls. FDG uptake in the left hippocampus (A) and the right hippocampus (B) was not significantly correlated with sTREM2 for AD patients, while the correlation was negative. Additionally, FDG uptake in the left hippocampus (C) and the right hippocampus (D) was not significantly correlated sTREM2 for controls. However, the correlation was positive though it did not reach a statistical significance.

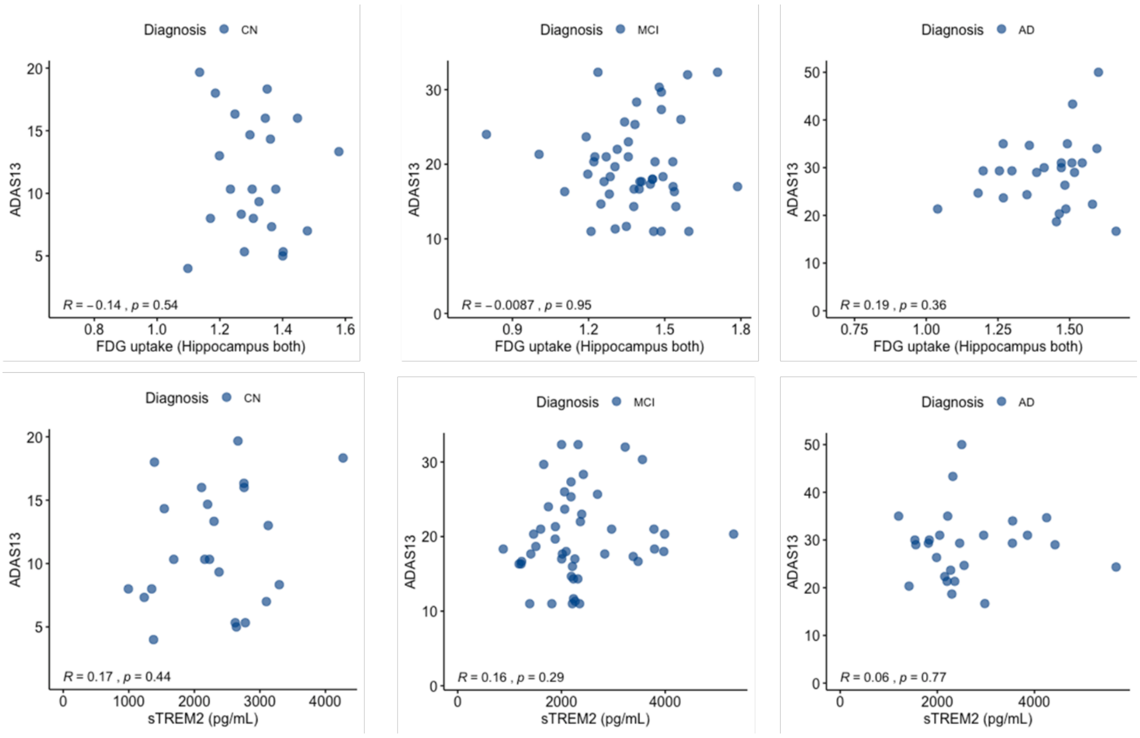

Supplemental figure 8. The correlation between the cognitive score and hippocampal FDG uptake. In the subgroups according to the diagnosis (CN, MCI and AD), hippocampal FDG uptake was not associated with the severity of a cognitive score, Alzheimer's disease assessment scale (ADAS13) (*above*). In addition, sTREM2 was also not correlated with ADAS13 in all subgroup (AD, MCI and CN) (*below*).
